# Aortic Valve Replacement: A Case Report Exploring the Psychological Impacts of Cardiac Surgery

**DOI:** 10.1002/ccr3.70410

**Published:** 2025-04-07

**Authors:** Eteesha Rao

**Affiliations:** ^1^ Newcastle University Medical School Newcastle‐Upon‐Tyne UK

**Keywords:** aortic valve replacement; psychosocial impact, holistic care, postoperative recovery, psychological burden, stress and surgery

## Abstract

With over 5000 successful aortic valve replacements (AVR) carried out across the UK, thousands of patients leave hospital physiologically and physically treated, with the expectation that they will gradually integrate back into their day‐to‐day routines. However, the psychological burden associated with the surgery is often underestimated. We describe a 55‐year‐old male attending a cardiology clinic with severe aortic regurgitation due to thickened aortic valve cusps. He was admitted for 9 days at a specialist hospital 2 h away from his home for AVR, requiring him to leave his wife for whom he is the sole carer. The burden of caregiving, coupled with the stress of surgery and post‐operative recovery, led to heightened psychological distress, impeding his recovery. The burden and stress were measured using the Short Form‐36 (SF‐36) health survey and self‐reported daily ratings of pain, physical discomfort, weakness, depression, and irritability. While most recover physically after open‐heart surgery, psychological distress can slow overall recovery. Stress and inadequate recovery are linked through physiological mechanisms, including heightened inflammatory responses and impaired immune function. The patient's well‐being and remission progression were assessed through follow‐up clinic visits and SF‐36 questionnaires. Importantly, these were not reported back to the GP upon hospital discharge, representing a gap in holistic recovery management. The discussion elaborates on the necessity of integrating psychological assessment in AVR recovery and how stress pathways affect long‐term outcomes. Recovery should not be focused solely on the physical aspects of surgery but also on the psychological and social impacts.

**Summary**

- Aortic valve replacement (AVR) improves physiological outcomes, but psychosocial factors significantly impact recovery.
- Patients often face stress about returning to normal life, caregiving responsibilities, and financial concerns.
- Ensuring healthcare professionals screen and address these issues through holistic preoperative planning and psychosocial support can enhance postoperative recovery and overall patient well‐being.

## 1 Introduction

Aortic valve replacement (AVR) is a surgical procedure used to treat conditions affecting the aortic valve, primarily aortic stenosis and aortic regurgitation. It involves removing the damaged valve and replacing it with a mechanical or biological prosthetic valve [1]. The valve type depends on patient factors such as age and lifestyle. The procedure is aimed at restoring normal blood flow out of the left ventricle into the aorta, reducing symptoms, improving quality of life, and survival [2].

AVR performed by cardiac surgeons is a type of major surgery, routinely carried out under general anesthesia, making a midline sternotomy incision from the sternal notch to the xiphisternum. The heart is stopped with its function taken over by a cardiopulmonary bypass machine during the operation. Surgical complications commonly include infections, bleeding risks, thrombosis, thromboembolic events that is, stroke, arrhythmia, and reduced renal function. Post‐operative recovery in the hospital typically takes 1 week, with the patient returning to normal activities after a few weeks and making a full recovery after 2–3 months [1].

With over 5000 successful aortic valve replacements (AVR) carried out across the UK, thousands of patients leave the hospital physiologically and physically treated. The expectation is that they will gradually, seamlessly integrate back into their day‐to‐day routines, but this is not always the case in reality [2, 3].

## 2 Case History and Examination

A 55‐year‐old male, working as an accountant, was referred by his general practitioner to a cardiology clinic with symptoms of chest pain, presyncope, and tiredness for the past 3 months. The patient had never smoked and had a past medical history of high cholesterol and a left‐sided stroke 6 years prior. He is the sole carer of his wife, who has complex mental health needs.

On examination, he had a BMI of 28.29 (overweight) and pitting oedema. His echocardiogram findings indicated that he had severe aortic regurgitation due to thickened aortic valve cusps with normal left ventricular size and function. The patient was referred to a tertiary centre, located 2 h from his place of residence, for consultation to undergo an AVR.

## 3 Differential Diagnoses

The history and examination findings for this patient are highly indicative of severe aortic regurgitation as confirmed by his transoesophageal echocardiogram findings. Other possible differential diagnoses for patients presenting with a three‐month history of symptoms of chest pain, presyncope, and tiredness could include aortic stenosis, acute coronary syndrome, hypertrophic obstructive cardiomyopathy, and pulmonary embolism.

## 4 Conclusion and Results

Although his post‐operative recovery was medically uneventful, he experienced significant psychological distress due to prolonged hospitalization, separation from his wife, and concerns about managing daily life post‐surgery. These factors contributed to delayed emotional and functional recovery. His psychological burden was assessed throughout his inpatient admission using the SF‐36 questionnaire. One of the eight sections of this questionnaire looks at psychological distress and well‐being.

## 5 Discussion

While most patients who undergo open‐heart surgery experience a relatively swift physical recovery, the psychosocial impacts of the procedure are often substantial and can interfere with overall wellbeing. Studies suggest that stress triggers prolonged inflammatory responses and impairs immune function, thereby delaying tissue healing and prolonging recovery. This highlights the direct physiological link between psychological distress and surgical outcomes.

Anxiety over other stressors such as work, finances, and the well‐being of dependents is often a significant factor in postoperative recovery. While support resources are available, they are not always well‐advertised or accessible to patients, leaving some to navigate this challenging time without adequate psychosocial support. Ben‐Zur et al. (2000) highlight how high psychological distress coupled with limited physical capacity often leads to ineffective emotion‐focused coping strategies, which can lower patients' quality of life and overall satisfaction with recovery [3]. This underscores the importance of acknowledging and addressing these broader concerns to foster a holistic recovery process.

Psychological pathways linking stress to cardiac surgery recovery include neuroendocrine responses, autonomic dysfunction, and behavioral factors. Chronic stress increases cortisol levels, which can impair immune responses and delay wound healing. Stress‐related activation of the sympathetic nervous system may contribute to heightened cardiovascular strain postoperatively [4]. Depression and anxiety may reduce motivation for rehabilitation, adherence to medication, and engagement in physical therapy. A study by Contrada et al. (2008) studied 550 patients undergoing heart surgery, including bypass grafting and valve procedures, and found that unresolved psychosocial issues and presurgical distress negatively impacted patients' adaptation to postoperative recovery. This study emphasizes that a more holistic approach, addressing both physical and psychosocial needs, could greatly improve patient outcomes [5]. Similarly, inadequate social support, as seen in this case, correlates with poorer outcomes. King et al. (1993) demonstrated that strong spousal and social support significantly improves patient outcomes after cardiac surgery, reinforcing the importance of assessing a patient's support system preoperatively [6].

The assessment of this patient's well‐being and remission progression was conducted through follow‐up clinic visits and the SF‐36 questionnaire. However, one critical oversight was the failure to report inpatient psychological scoring tests back to the GP upon hospital discharge. This omission represents a gap in long‐term recovery planning, as mental health assessments should be incorporated into primary care follow‐up.

To improve patient outcomes, healthcare professionals should implement preoperative psychological screening by identifying high‐risk patients through validated tools like SF‐36. Enhanced discharge planning should ensure that both physical and psychological recovery metrics are reported to GPs on discharge to allow for appropriate follow‐up and community support plans. Psychosocial interventions should provide access to counseling, caregiver support, and social work services.

Postoperative recovery should encompass both physical and psychological well‐being. This case highlights the necessity of integrating psychosocial factors into the recovery plan to optimize outcomes. While based on a single case, the findings align with broader literature emphasizing the role of psychological distress in cardiac surgery recovery. Future approaches should incorporate structured psychological assessments and support systems to enhance holistic recovery and ensure these are passed on to primary care professionals to enable continuity of care.

Post‐surgical recovery should not be focused solely on the physicality of the surgery but also the psychological and social impact on the patient post‐surgery. Our case highlights the importance of recovery plans being ‘patient‐defined’ rather than clinically defined [7]. It needs to be acknowledged that the outcomes here are based on a single case, however the results align with the body of literature and illuminate an important aspect for consideration in planning for the holistic recovery of patients.

## Author Contributions

**Eteesha Rao:** conceptualization, data curation, formal analysis, methodology, writing – original draft, writing – review and editing.

## Consent

Written, informed, and voluntary consent was taken from the patient prior to writing up this case report. The patient is aware that their case will be used for research purposes and has consented for this to be published.

## Conflicts of Interest

The author declares no conflicts of interest.

## Data Availability

Data sharing not applicable to this article as no datasets were generated or analyzed during the current study.
